# Influence of Healthy Plant‐Based Fat Substitutes on the Sensory and Nutritional Properties of Oat‐Date Balls

**DOI:** 10.1002/fsn3.71193

**Published:** 2025-11-21

**Authors:** Nashwa M. Younes, Mahmoud Younis, Mahmoud Khalil, Diaeldin Omer Abdelkarim, Samar M. S. Shawir

**Affiliations:** ^1^ Home Economics Department, Faculty of Specific Education Alexandria University Alexandria Egypt; ^2^ Chair of Dates Industry and Technology King Saud University Riyadh Saudi Arabia; ^3^ Institute of Nutritional Science, Faculty of Science Potsdam University Potsdam Germany; ^4^ Department of Agricultural Engineering, Faculty of Engineering University of Khartoum Khartoum Sudan

**Keywords:** date balls, fat substitutes, healthy, nutritional properties, sensory evaluation

## Abstract

Snack bars and date‐based energy products have gained increasing popularity as convenient, nutrient‐dense options that cater to the growing demand for functional foods among health‐conscious consumers. The present study aimed to develop oat semi‐dry date balls with healthy plant‐based fat substitutes, peanut butter (PB), coconut butter (CB), and sesame butter (SB), and evaluate their effects on nutritional composition, antioxidant activity, physical properties, and sensory attributes. The proximate analysis revealed significant enhancements in macronutrient composition across the fat‐substituted samples. Date balls‐PB significantly increased protein content (14.4 g/100 g), while Date balls‐CB contributed the highest fat content (30.7 g/100 g) and caloric value (538.9 kcal/100 g). Date balls‐SB notably improved the mineral profile, particularly iron (6.21 mg/100 g), calcium (242.4 mg/100 g), and magnesium (162.5 mg/100 g). All treatments exhibited superior antioxidant potential compared to the control, as indicated by elevated total phenolic and flavonoid contents and enhanced DPPH and ABTS radical scavenging activities. Texture analysis indicated a reduction in hardness and gumminess, especially in the Date balls‐CB formulation, which resulted in improved palatability. Sensory attributes showed that Date balls‐PB and Date balls‐CB formulations achieved the highest acceptability scores across all sensory attributes, including flavor, texture, and overall preference.

## Graphical abstract

Schematic overview of the formulation of oat–date balls enriched with peanut, coconut, and sesame butters and the subsequent evaluation of their antioxidant activity, total phenolic and flavonoid contents, proximate composition, texture, fatty acid profile, and sensory attributes.

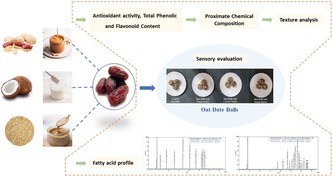

## 1 Introduction

Dates ( *Phoenix dactylifera* L.) are among the most widely consumed fruits worldwide and represent a crop of great economic and cultural importance in Egypt, which is the world's largest producer of dates (Al‐Karmadi and Okoh 2024). Owing to their rich nutritional profile, dates are considered a functional food, providing natural carbohydrates, dietary fibers, proteins, essential minerals, and bioactive compounds with antioxidant potential (Soomro et al. 2023). In particular, Siwi dates cultivated in the Siwa Oasis are highly valued for their high sugar content, phenolic compounds, and minerals such as potassium, magnesium, and calcium. Previous studies have attributed their health‐promoting properties to strong antioxidant activity, which helps mitigate oxidative stress and chronic disease risk (Abdelbaky et al. 2023; Ahmed et al. 2014; Manai et al. 2024).

Increasing public attention has been directed toward improving health and well‐being through the consumption of wholesome and functional foods. Grains containing oats have been widely recognized for their beneficial effects on human health and their ability to reduce the risk of certain diseases (Sterna et al. 2016). Oat‐based products have attracted considerable attention for their multiple health benefits, including weight management, reduction of postprandial glycemia, and lowering of serum low‐density lipoprotein (LDL) cholesterol (Dong et al. 2016; Rasane et al. 2015). These physiological benefits are mainly attributed to β‐glucan, a non‐starch polysaccharide present in significant amounts in oats, which exhibits lipid‐lowering, insulin‐modulating, and glycemic‐regulating properties (Peng et al. 2013; Wood 2010). Moreover, oats are a rich source of dietary fiber, high‐quality protein, and complex carbohydrates that contribute to lowering cholesterol levels and improving digestive health. Their balanced nutrient composition and functional properties make them an excellent ingredient for energy‐based snack formulations (Tang et al. 2023). Therefore, oats were incorporated in the present study to enhance the nutritional profile, texture, and overall health‐promoting potential of the developed energy balls enriched with plant‐based fat substitutes.

With the global shift in eating habits and heightened consumer awareness of diet‐related health concerns, demand for functional foods has grown significantly. Consumers increasingly prioritize products enriched with natural bioactives, improved nutritional value, and better sensory quality (Birch and Bonwick 2019). In this context, date‐based snack products such as energy balls and bars have emerged as convenient and nutrient‐dense options for both daily consumption and sports nutrition. The integration of dried fruits, nuts, and plant‐derived fats further enhances the antioxidant potential and health‐promoting value of these products (Ali Haimoud et al. 2016; Marangoni et al. 2019).

Plant‐based fats are gaining special attention as healthier alternatives to conventional animal fats due to their richness in essential fatty acids, vitamins, minerals, and bioactive compounds. Peanut ( *Arachis hypogaea* L.) provides a high proportion of unsaturated fatty acids and proteins, in addition to antioxidant phytochemicals (Akram et al. 2018). Coconut ( *Cocos nucifera* L.) is a valuable source of medium‐chain triglycerides that are easily metabolized and associated with improved satiety, energy balance, and oxidative stability (Duranova et al. 2025; Lu et al. 2024). Sesame ( *Sesamum indicum* L.), known as the “queen of oilseeds,” contributes bioactive lignans such as sesamol and sesamin, along with flavonoids and phenolics that offer antioxidant and anti‐inflammatory benefits (Sharma et al. 2020; Wei et al. 2022). Incorporating these fats into functional snack formulations can thus enhance nutritional quality and sensory acceptability while offering health‐promoting effects.

Despite the recognized value of dates and plant‐derived fats, there is still limited research on combining different healthy fat substitutes with semi‐dry oat–date formulations and evaluating their collective influence on nutritional, bioactive, physical, and sensory properties. This study is therefore designed to prepare oat–date balls enriched with peanut butter, coconut butter, and sesame butter as plant‐based fat alternatives to dairy butter. The objective is to compare their impact on proximate composition, mineral content, antioxidant potential, fatty acid profile, texture, and sensory acceptance. The present study aimed to prepare date balls enhanced with different plant‐based fats (peanut butter, coconut butter, and sesame butter) and evaluate their effects on the sensory properties and nutritional value. The novelty of this work lies in providing a comprehensive assessment of how different plant‐based fats uniquely modulate the health‐promoting and consumer‐oriented attributes of date‐based snacks, thereby offering a scientific basis for developing next‐generation functional snack foods targeted at health‐conscious consumers and athletes.

## 2 Materials and Methods

### 2.1 Samples

Siwi semi‐dry dates ( *Phoenix dactylifera* L.) were obtained from a private factory in Siwi, Matrouh Governorate, Egypt. Oat flakes ( *Avena sativa* ), peanut butter, coconut butter, and sesame butter, in addition to all ingredients used in the preparation of oat‐date balls, were purchased from local markets (Carrefour, Alexandria, Egypt).

### 2.2 Preparation of Siwi Semi‐Dry Date Paste

The dates were cleaned to remove any surface dust or impurities, then manually pitted. After that, they were thoroughly minced using a domestic electric grinder (Moulinex, France) to obtain a smooth, uniform date paste suitable for mixing with the other ingredients. The ground forms were placed in sealed glass containers and kept in a refrigerator at 4°C until further analysis.

### 2.3 Preparation of Semi‐Dry Date Balls Enriched With Healthy Fat Substitutes

**FIGURE 1:**
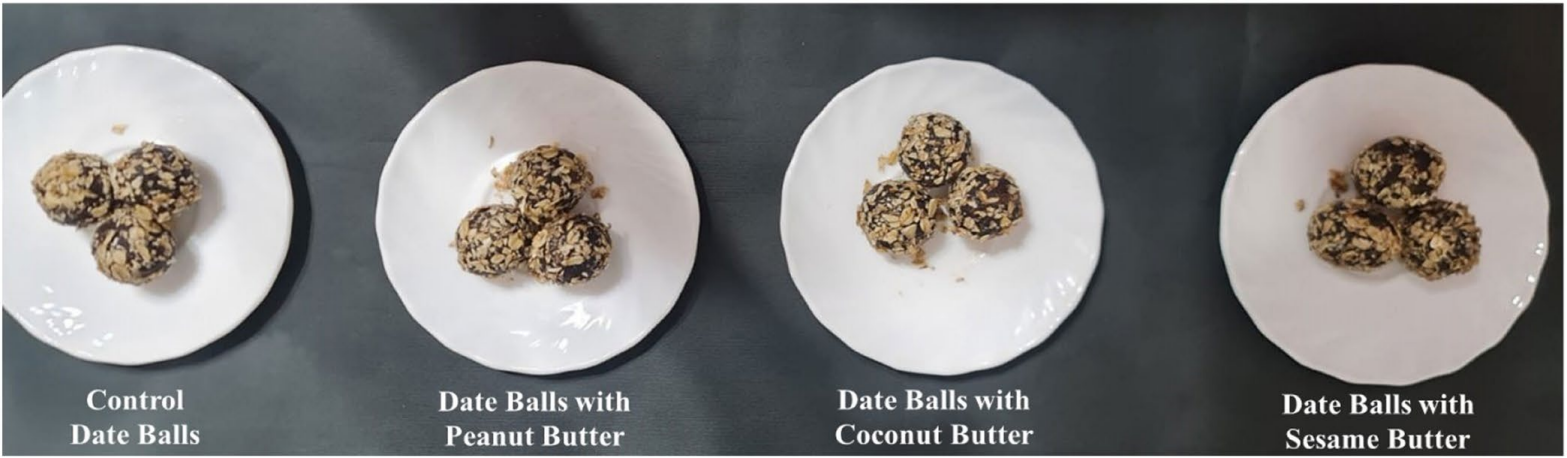
Sensory evaluation of the oat‐date balls with healthy plant‐based fat substitutes.

Date balls were formulated using mixtures (20%) of natural fat sources, including peanut butter, coconut butter, and/or sesame butter as healthy fat substitutes. A control group containing dairy butter was also prepared for comparison (Table 1 and Figure 1). In a clean bowl, oat flakes and pitted mashed dates were blended with plant‐based fat sources. The mixture was kneaded manually for approximately 5 min until a sticky, uniform mass was obtained. The dough was then shaped into small, round balls by hand, with a diameter of approximately 2 cm. The formed balls were placed on trays and left to rest at room temperature for 1 h to stabilize their structure. Subsequently, the samples were packed in polyethylene bags, hermetically sealed, and stored in airtight containers at a refrigeration temperature of 4°C until analysis.

**TABLE 1:** Formulation ingredients of semi‐dry date balls enriched with healthy plant‐based fat substitutes.

Ingredients	Control Date Balls	Date Balls with (PB)	Date Balls with (CB)	Date Balls with (SB)
Oat flakes	10	10	10	10
Date paste	70	70	70	70
Fat source	20*	20	20	20
Total weight (g)	100	100	100	100

Abbreviations: CB, Coconut Butter; PB, Peanut Butter; SB, Sesame Butter.

* Dairy butter. * Statistically significant difference at *p* < 0.05 (Duncan’s multiple range test).

### 2.4 Determination of Proximate Analysis

The moisture, protein, fat, and ash contents of date balls formulated with healthy‐based fat alternatives were analyzed following the official methods of AOAC (2005). Total carbohydrate content was determined in accordance with Chen et al. (2013), while total sugars were quantified according to the procedures described by AOAC (2005).

Energy values (kcal/100 g) were estimated using the formula:

$$\text{Calories}=\left(9\times \%\mathrm{fat}\right)+\left(4\times \%\text{protein}\right)+\left(4\times \%\text{carbohydrate}\right)$$

Total dietary fiber (TDF) was determined using an ANKOM 220 Fiber Analyzer (ANKOM Technology, USA) following the AOAC enzymatic–gravimetric method 991.43/985.29: ~1 g ground sample (defatted if needed) was sequentially hydrolyzed with heat‐stable α‐amylase (95°C–100°C), protease (60°C, pH 7.5), and amyloglucosidase (60°C, pH 4.5); the residue was filtered in F57 bags, washed with hot water and acetone, dried (105°C), then corrected for ash (525°C) and protein (*N* × 6.25). Results are reported as % TDF (dry basis, mean ± SD) with blanks and a certified control (AOAC 2004).

### 2.5 Antioxidant Activity Assays

#### 2.5.1 DPPH Radical Scavenging Assay

The DPPH radical scavenging activity of date balls formulated with healthy‐based fat was determined according to Wronkowska et al. (2010), with minor modifications. An aliquot of 20 μL of each sample extract was mixed with 180 μL of 0.1 mM DPPH solution in methanol in a 96‐well microplate. The mixture was incubated in the dark at room temperature for 20 min, and absorbance was measured at 517 nm. The scavenging activity was calculated as:

$$\text{DPPH}\cdot \text{scavenging activity}\;\left(\%\right)=\left[\left(A_{0}-A_{1}\right)/A_{0}\right]\times 100$$

where $A_{0}$ is the absorbance of the control (blank) and $A_{1}$ is the absorbance of the sample. All measurements were performed in triplicate.

#### 2.5.2 ABTS Radical Cation Decolorization Assay

Antioxidant activity of date balls formulated with healthy‐based fat was evaluated using the ABTS assay (Altuncevahir et al. 2024) in a 96‐well format. The ABTS•^+^ radical cation was generated by reacting 7 mM ABTS with 2.45 mM potassium persulfate and allowing the mixture to stand in the dark for 12–16 h. The resulting solution was diluted with ethanol to an absorbance of 0.70 ± 0.02 at 734 nm. A 20 μL aliquot of each sample or Trolox standard was mixed with 170 μL of the ABTS•^+^ working solution, incubated for 6 min at 30°C, and the absorbance was recorded.

### 2.6 Determination of Bioactive Compounds

#### 2.6.1 Total Phenolic Content

Total phenolic content of date balls formulated with healthy‐based fat was determined using the Folin–Ciocalteu and aluminum chloride colorimetric methods (Debnath and Manna 2019). Results were expressed as gallic acid equivalents (GAE) per 100 g dry weight.

#### 2.6.2 Total Flavonoid Content

Total flavonoid content of date balls formulated with healthy‐based fat was measured using catechin as the standard. Absorbance was recorded at 517 nm, and results were expressed as catechin equivalents (CE) per 100 g dry weight (Meda et al. 2005).

### 2.7 Mineral Content

The mineral composition (Fe, Ca, Mg, K, Zn, and Mn) of date balls formulated with healthy‐based fat substitutes was determined on a dry‐weight basis using an atomic absorption spectrophotometer (Shimadzu, model AA‐6650) following the method of Al‐Showiman (1990).

### 2.8 Determination of Physical Properties

Hardness, adhesiveness, springiness, gumminess, and chewiness of the date balls formulated with healthy‐based fat substitutes were measured using a Brookfield Texture Analyzer. A fixed amount of each sample was placed in a plastic container (~50 mm depth) to achieve uniform thickness. A cylindrical probe (TA4/1000, 38 mm diameter) was used to measure the compression force. Samples were compressed to a depth of 20 mm at a speed of 1 mm/s with a trigger load of 5 g. All measurements were conducted at a controlled room temperature (25°C) following the method of Singh et al. (2015).

### 2.9 Fatty Acid Profile Analysis by Gas Chromatography

#### 2.9.1 Oil Extraction and Preservation

Approximately 10 g of date balls containing healthy‐based fat substitutes (peanut butter, coconut butter, and sesame butter) were subjected to oil extraction using petroleum ether (40°C–60°C) in a Soxhlet apparatus. Extraction conditions included a 30 min immersion period with the thimble submerged in boiling solvent, followed by 60 min of reflux washing. Solvent removal was performed using a rotary evaporator, after which the extracted oil was flushed with nitrogen and stored at −20°C until analysis (Jajić et al. 2013).

#### 2.9.2 Gas Chromatographic Analysis of Fatty Acids

Fatty acid profiles were determined following lipid transmethylation to fatty acid methyl esters (FAMEs) using the modified method of Liu (1994). Analyses were performed using an HP 6890 Plus gas chromatograph (Hewlett Packard, USA) equipped with a flame ionization detector (FID) and a Supelco SP‐2380 capillary column (60 m × 0.25 mm × 0.20 μm, Sigma‐Aldrich, USA). Injector and detector temperatures were maintained at 250°C. The oven program was set as follows: an initial temperature of 140°C (held 5 min), increased to 240°C at 4°C/min, and held at 240°C for 10 min. Helium served as the carrier gas at 1.2 mL/min constant flow. A 1 μL sample in *n*‐hexane was injected in split mode (split ratio 100:20). FAMEs were identified by comparing retention times with those of an authentic 37‐component FAME standard mix (Supelco). Fatty acid composition was expressed as the relative percentage of the total identified peak areas.

### 2.10 Sensory Evaluation

Sensory evaluation of the semi‐dry date balls enriched with healthy‐based fat substitutes (peanut butter, coconut butter, and sesame butter) was conducted with 40 semi‐trained panelists from the Faculty of Specific Education, Alexandria University. Samples were evaluated for appearance, color, aroma, texture, taste, and overall acceptability using a 9‐point hedonic scale. The sensory evaluation was carried out at room temperature (approximately 25°C). Each panelist received one piece (about 10 g) of each sample, coded with random three‐digit numbers. Water was used between samples during tasting. The evaluation was conducted under normal lighting conditions in a quiet environment according to the method of Chunilal and Sudhakar (2021).

### 2.11 Statistical Analysis

Data were analyzed using IBM SPSS Statistics software, version 20.0 (IBM Corp., Armonk, NY, USA). Quantitative results were expressed as mean ± SD. Differences among groups were evaluated using one‐way analysis of variance (ANOVA), followed by the least significant difference (LSD) post hoc test. Statistical significance was considered at *p* < 0.05.

## 3 Result and Dissociation

### 3.1 Proximate Chemical Composition of Formulated Semi‐Dry Date Balls

The proximate chemical composition of semi‐dry date balls enriched with healthy plant‐based fat substitutes (peanut butter, coconut butter, and sesame butter) is presented in Table 2. Significant variations were observed in moisture, protein, fat, ash, carbohydrates, total sugars, and caloric content among the formulations. Protein content was notably higher in the peanut butter and sesame butter groups (14.4 g and 11.6 g/100 g, respectively) compared to the control (8.43 g/100 g). In contrast, coconut butter did not significantly alter protein levels relative to the control. Fat content increased markedly in all formulations compared to the control (4.6 g/100 g), with the highest value recorded in the coconut butter group (30.7 g/100 g), followed by peanut butter (25.0 g/100 g) and sesame butter (22.32 g/100 g). Dietary fiber content did not differ significantly among the samples, with values ranging from 4.32 to 5.5 g/100 g, suggesting that fiber retention was not affected by fat substitution. However, carbohydrate and total sugar contents decreased significantly in the fat‐enriched formulations. The control sample exhibited the highest carbohydrate (62.07 g/100 g) and sugar (59.78 g/100 g) contents, while the lowest were observed in the coconut butter group (43.0 g and 40.75 g/100 g, respectively), indicating the dilution effect of fat‐rich ingredients.

**TABLE 2:** Proximate chemical composition of oat semi‐dry date balls enriched with healthy plant‐based fat substitutes.

Formulation Analysis	Control‐DB	Date Balls‐PB	Date Balls‐CB	Date Balls‐SB	*p*	LSD 5%
Moisture (g)	17.9^a^ ± 2.95	7.90^b^ ±0.10	9.78^b^ ±0.09	8.60^b^ ±0.04	< 0.001*	2.776
Protein (g/100 g)	8.43^b^ ±0.81	14.40 ^a^ ± 1.40	10.20^b^ ± 2.16	11.60^a^ ± 1.78	0.011*	3.044
Fat (g)	4.60^c^ ±0.05	25.00^b^ ± 2.28	30.70^a^ ±0.98	22.32^b^ ± 2.55	< 0.001*	4.274
Ash (g)	1.70^a^ ± 0.05	1.30^c^ ± 0.08	1.50^b^ ± 0.002	1.42^b^ ± 0.07	< 0.001*	0.126
Dietary Fiber	5.30^a^ ± 0.40	5.50^a^ ± 0.59	4.70^a^ ± 0.72	4.32^a^ ± 0.79	0.176	1.215
Carbohydrate (g)	62.07^a^ ± 4.52	45.58^c^ ± 2.08	43.00^c^ ± 2.60	52.34^b^ ± 3.77	0.001*	6.367
Total Sugars (g)	59.78 ^a^ ± 2.38	42.30^c^ ± 1.09	40.75^c^ ± 1.45	49.62^b^ ± 1.26	< 0.001*	3.052
Calories (Kcal)	323.4^c^ ± 6.04	508.7^b^ ± 9.57	538.9^a^ ± 10.83	493.3^b^ ±7.07	< 0.001*	16.177

*Note:* Values within the same row sharing different letters are significantly different. Data are presented as mean ± SD, based on three replicates per group.

Abbreviations: CB, Coconut Butter; DB, Date Balls; PB, Peanut Butter; SB, Sesame Butter.

A significant decrease in total sugar content was observed in all formulations containing plant‐based fat substitutes compared with the control. This reduction may be considered nutritionally advantageous for consumers seeking products with lower simple carbohydrate content. However, this benefit was accompanied by an increase in total fat and caloric values, particularly in samples containing peanut and coconut butter. Similar findings were reported by X. Peng and Yao (2017), who noted that replacing carbohydrate‐based ingredients with plant fats can improve the lipid profile but also raise the overall energy density of the product. At the same time, nut‐based lipid sources (e.g., peanut products) tend to improve blood‐lipid profiles—lowering total and LDL cholesterol and, in hypertriglyceridemic subjects, triglycerides—in a dose‐responsive manner across multiple controlled trials and pooled analyses (Guasch‐Ferre et al. 2023; Sabaté et al. 2010). These observations justify interest in plant‐fat formulations that reduce simple sugars while emphasizing unsaturated fatty acids (MUFA/PUFA) and transparent serving guidance. In parallel, structured plant‐oil systems (oleogels/bigels) are an emerging strategy to retain unsaturated‐fat quality while improving techno‐functionality in baked snacks—potentially helping manage energy density at the product level (Silva, da Silva et al. 2023). For context, the carbohydrate‐as‐fat‐replacer framework summarized by X. Peng and Yao (2017) explains why such reformulations can shift both texture and caloric density.

Caloric content increased significantly with fat substitution, with the coconut butter group exhibiting the highest energy value (538.9 kcal/100 g), followed by peanut butter (508.7 kcal/100 g) and sesame butter (493.3 kcal/100 g), compared to the control (323.4 kcal/100 g).

These findings are consistent with earlier studies that examined the nutritional impact of nut‐ and seed‐based fat incorporation. The sharp rise in fat content, particularly in the coconut butter group, corroborates the observations of Amadi et al. (2025), who reported that coconut paste significantly enhanced lipid content in functional candies. Similarly, the substantial protein enrichment achieved with peanut and sesame butter aligns with the results of Ayo‐Omogie (2023), who demonstrated that peanut and sesame pastes are rich protein sources that improve the nutritional value of composite baked products.

The reduction in moisture across all fat‐substituted formulations compared to the control agrees with Mehta and Pinto (2023), who reported that fat‐rich ingredients often displace water in formulations, lowering moisture content and potentially enhancing shelf‐life. In terms of carbohydrate and sugar reduction, our results confirm the findings of Gorrepati et al. (2015), who highlighted that the addition of nut or seed butters decreases carbohydrate density due to their inherently lower sugar and starch contents.

Regarding fiber content, no significant differences were observed between treatments in the current study. This observation contrasts with Colla et al. (2018), who reported fiber enrichment when nut pastes were incorporated into bakery formulations. The discrepancy may be attributed to differences in the fiber contribution of the specific fat substitutes used in this work.

Alsuhebani et al. (2025) improved date snacks primarily via protein‐focused fortification (Samh seed + milk proteins), whereas our advance comes from lipid‐phase optimization (peanut/sesame/coconut butters) that shifts the fatty‐acid profile and mouthfeel through different techno‐functional pathways. For instance, Al‐Hooti et al. (1997) showed that nut/seed enrichment upgrades sensory quality and macronutrients in date bars; our data extend this line by pinpointing which butter source delivers the most favorable balance (peanut/sesame over coconut) and by quantifying formulation‐dependent trade‐offs to guide targeted product positioning.

Finally, the elevated caloric values in the fat‐substituted formulations, particularly coconut butter (538.9 kcal/100 g), are in strong agreement with Drewnowski (2003), who emphasized that dietary fat is the most energy‐dense macronutrient, substantially raising the caloric density of food products.

Taken together, these results highlight the functional potential of plant‐based fat incorporation into date‐based snacks. While each fat source contributed distinct nutritional advantages, the peanut butter formulation exhibited the most balanced macronutrient profile, combining the highest protein content with moderate fat levels and lower sugar content. This makes it the most nutritionally beneficial formulation among the evaluated samples, complementing the observations of previous studies on nut‐ and seed‐enriched foods.

### 3.2 Mineral Content of Formulated Oat Semi‐Dry Date Balls

The mineral composition of semi‐dry date balls enriched with plant‐based fat substitutes (peanut butter, coconut butter, and sesame butter) is shown in Table 3. Sesame butter markedly enhanced most minerals compared with the control, except potassium. The highest iron concentration was observed in the sesame butter group (6.21 mg/100 g), followed by coconut butter (4.51 mg/100 g), while the control had the lowest (2.43 mg/100 g). Calcium and magnesium were also the greatest in sesame butter samples (242.4 mg and 162.5 mg/100 g, respectively) compared with the control (63.12 mg and 66.24 mg/100 g). Similarly, zinc and manganese contents peaked in sesame butter (3.26 and 2.58 mg/100 g), relative to the control (0.78 and 0.68 mg/100 g). Conversely, potassium was highest in the control (644.3 mg/100 g) and declined in all fat‐substituted groups.

**TABLE 3:** Mineral content of oat semi‐dry enriched with healthy plant‐based fat substitutes.

Samples (mg/100 g)	Control‐DB	Date Balls‐PB	Date Balls‐CB	Date Balls‐SB	*p*	LSD 5%
Iron	2.43^c^ ± 0.17	3.03^c^ ± 0.10	4.51^b^ ± 0.08	6.21^a^ ± 0.61	< 0.001*	0.602
Calcium	63.12^b^ ± 5.96	55.7^bc^ ± 4.62	50.21^c^ ± 2.43	242.4^a^ ± 8.21	< 0.001*	10.738
Magnesium	66.24^d^ ± 4.62	126.12^b^ ± 7.92	98.91^c^ ± 8.60	162.5^a^ ± 5.66	< 0.001*	12.980
Potassium	644.3^a^ ± 19.01	563.82^b^ ± 17.39	505.41^c^ ± 10.18	527.87^c^ ± 16.27	< 0.001*	30.245
Zinc	0.78^d^ ± 0.08	2.37^b^ ± 0.12	1.87^c^ ± 0.19	3.26^a^ ± 0.06	< 0.001*	0.231
Manganese	0.68^c^ ± 0.06	2.37^b^ ± 0.19	2.32^b^ ± 0.04	2.58^a^ ± 0.01	< 0.001*	0.190

*Note:* Values within the same row sharing different letters are significantly different. Data are presented as mean ± SD, based on three replicates per group.

Abbreviations: CB, Coconut Butter; DB, Date Balls; PB, Peanut Butter; SB, Sesame Butter.

These results demonstrate a significant increase (*p* < 0.001) in essential minerals (Ca, Mg, Fe, Zn, Mn) in formulations with sesame butter, followed by coconut and peanut butter. The elevated calcium and magnesium in sesame butter agree with Om et al. (2020), who reported sesame as a rich source of bioavailable minerals. Increased iron and zinc in the coconut butter group are consistent with Pandiselvam et al. (2024), who highlighted the mineral contribution of coconut‐based products. The higher potassium level in the control aligns with M Abo‐El‐Saad and MS Shawir (2024), who noted that semi‐dry dates are naturally potassium‐rich. Overall, these findings confirm that incorporating sesame, coconut, and peanut butters significantly improves the mineral profile of date balls, thereby enhancing their nutritional value.

### 3.3 Antioxidant Activity, Total Phenolic and Flavonoid Content of Formulated Semi‐Dry Date Balls

The antioxidant activity and bioactive compound contents of semi‐dry date balls enriched with plant‐based fats are shown in Table 4. All enriched samples exhibited significantly higher antioxidant activity and phenolic content than the control. DPPH scavenging activity ranged from 54.81 to 56.85 μmol TE/g in the fat‐substituted groups versus 43.59 μmol TE/g in the control, with slightly lower IC₅₀ values in sesame (2.71 mg/mL) and peanut butter (2.81 mg/mL) samples compared to the control (3.22 mg/mL). ABTS activity followed a similar trend, highest in the peanut butter group (59.53 μmol TE/g) and lowest in the control (45.44 μmol TE/g). Although IC₅₀ differences were not always significant, lower values generally reflected improved antioxidant efficiency, consistent with Wojdyło et al. (2007), who reported that phenolic‐rich foods exhibit stronger radical scavenging capacity.

**TABLE 4:** Antioxidant activity (DPPH and ABTS), ABTS, total phenolic, and flavonoid content of oat semi‐dry date enriched with healthy plant‐based fat substitutes.

Antioxidant and Bioactive assay	Control‐DB	Date Balls‐PB	Date Balls‐CB	Date Balls‐SB	*p*	LSD 5%
DPPH (μmol TE/g)	43.59 ^b^ ± 2.28	56.85 ^a^ ± 3.63	54.88 ^a^ ± 4.22	54.81 ^a^ ± 3.63	0.006*	6.613
IC_50_ (mg/g)	3.22 ^a^ ± 0.27	2.81^b^ ± 0.18	2.91 ^ab^ ± 0.23	2.71^b^ ± 0.18	0.091	0.410
ABTS (μmol TE/g)	45.44 ^c^ ± 1.16	59.53^a^ ± 2.02	52.99 ^b^ ± 2.31	58.08 ^ab^ ± 4.77	0.001*	5.447
Total phenolic (mg/g)	219.2 ^c^ ± 9.33	296.91 ^a^ ± 7.24	260.23 ^c^ ± 5.79	280.2 ^b^ ± 7.84	< 0.001*	14.413
Total Flavonoid content (mg/g)	28.01^c^ ± 1.73	33.45 ^ab^ ± 1.14	31.0 ^b^ ± 2.01	35.32 ^a^ ± 3.10	0.014*	3.989

*Note:* Values within the same row sharing different letters are significantly different. Data are presented as mean ± SD, based on three replicates per group.

Abbreviations: CB, Coconut Butter; DB, Date Balls; PB, Peanut Butter; SB, Sesame Butter.

Total phenolic content was greatest in the peanut butter group (296.91 mg/mL), followed by sesame butter (280.2 mg/mL), while the control recorded the lowest value (219.2 mg/mL). Flavonoid content peaked in sesame butter (35.32 mg/mL) versus the control (28.01 mg/mL), with intermediate levels in peanut and coconut treatments (33.45 and 31.0 mg/mL). These findings agree with Nemli et al. (2025), who highlighted that peanuts, sesame, and coconut enhance antioxidant potential due to their phenolics and flavonoids, and with Pokorný and Parkányiová (2019), who showed that oats and sesame are rich in phenolic acids that improve radical scavenging activity. Moreover, the strong flavonoid contribution of sesame aligns with Ahmad and Ghosh (2020), who emphasized sesamol and sesamin as key antioxidant lignans. The slightly weaker correlation between phenolic content and antioxidant activity observed here echoes the findings of Jacobo‐Velázquez and Cisneros‐Zevallos (2009) and may be explained by the presence of lipophilic antioxidants in lipid‐rich matrices, such as tocopherols and resveratrol, as discussed by Charlton et al. (2023).

Overall, enrichment with peanut, coconut, or sesame butters significantly enhanced the antioxidant properties of oat–date balls, with peanut butter contributing most to phenolics, and sesame butter showing the highest flavonoid enrichment.

### 3.4 Texture Analysis of Formulated Semi‐Dry Date Balls

Table 5 shows the texture profile analysis of oat–date balls enriched with plant‐based fat substitutes. All treatments exhibited a significant reduction in hardness compared with the control (420 g), with the lowest value in the coconut butter sample (270 g), followed by peanut butter (300 g) and sesame butter (310 g). This indicates a pronounced softening effect from fat incorporation. Gumminess was similarly reduced, especially in coconut butter samples (120 g vs. 150 g in the control). Chewiness values ranged from 84.2 to 99.8 mJ; sesame butter showed the highest value, slightly above the control, while coconut butter recorded the lowest. No significant differences were observed in adhesiveness or springiness across treatments.

**TABLE 5:** Texture analysis of oat semi‐dry date balls enriched with healthy plant‐based fat substitutes.

Samples	Texture Analysis				
	Hardness (g)	Adhesiveness (mj)	Springiness (mm)	Gumminess (g)	Chewiness (mj)
Control DB	420 ^a^ ± 8.33	0.16^b^ ± 0.02	0.65^b^ ± 0.03	150^a^ ± 7.09	97.4^a^ ± 5.76
Date Balls‐PB	300^b^ ± 10.83	0.19^ab^ ± 0.03	0.72^ab^ ± 0.06	130^bc^ ± 7.12	93.7^a^ ± 6.47
Date Balls‐CB	270 ^c^ ± 13.04	0.20^a^ ± 0.04	0.73^ab^ ± 0.05	120^c^ ± 5.15	84.2^b^ ± 3.02
Date Balls‐SB	310^b^ ± 8.26	0.18^ab^ ± 0.01	0.75^a^ ± 0.04	135^b^ ± 3.11	99.8^a^ ± 2.46
*p*	< 0.001*	0.072	0.102	0.002*	0.017*
LSD 5%	19.404	0.030	0.083	11.030	8.938

*Note:* Values within the same column sharing different letters are significantly different. Data are presented as mean ± SD, based on three replicates per group.

Abbreviations: CB, Coconut Butter; DB, Date Balls; PB, Peanut Butter; SB, Sesame Butter.

Coconut butter contributed to the lowest hardness, gumminess, and chewiness, producing a lighter texture and enhanced mouthfeel. This finding is consistent with Mat et al. (2022), who linked medium‐chain fatty acids in coconut oil to improved product softness and consumer acceptance. Sesame butter samples showed greater elasticity (0.75 mm), which may be attributed to their protein and structural fiber content (Gabal 2024). Both coconut and peanut butter formulations also exhibited higher viscosity, likely due to their creamy and homogeneous structures, which support a smooth texture without excessive stickiness. These outcomes align with Gorrepati et al. (2015), who reported that nut and seed butters enhance the mechanical and sensory attributes of snack formulations.

### 3.5 Sensory Evaluation of Formulated Semi‐Dry Date Balls

Table 6 and Figure 1 present the sensory scores of oat–date balls enriched with plant‐based fat substitutes. All enriched formulations achieved significantly higher ratings than the control across all sensory attributes. Peanut butter and coconut butter samples received the highest overall acceptance scores (8.8 and 8.6, respectively), excelling in color, taste, odor, and texture. Sesame butter also demonstrated good acceptance (8.1), though slightly lower, likely due to its stronger, distinct flavor profile. The control consistently scored the lowest, confirming the positive role of plant‐based fats in enhancing sensory quality.

**TABLE 6:** Sensory evaluation of semi‐dry date balls enriched with healthy plant‐based fat substitutes.

Samples	Color	Taste	Odor	Texture	General acceptance
Control DB	7.0 ^c^ ± 0.13	7.1 ^c^ ± 0.14	6.7 ^c^ ± 0.19	7.2 ^c^ ± 0.13	6.2 ^c^ ± 0.17
Date Balls‐PB	8.01 ^b^ ± 0.19	8.8 ^a^ ± 0.15	8.3 ^a^ ± 0.18	8.6 ^a^ ± 0.12	8.8 ^a^ ± 0.18
Date Balls‐CB	8.4 ^a^ ± 0.20	8.6 ^a^ ± 0.18	8.2 ^a^ ± 0.20	8.4 ^a^ ± 0.20	8.6 ^a^ ± 0.19
Date Balls‐SB	8.3 ^ab^ ± 0.21	8.3 ^b^ ± 0.16	7.3 ^b^ ± 0.17	7.7 ^b^ ± 0.14	8.1 ^b^ ± 0.16
*p*	< 0.001*	< 0.001*	< 0.001*	< 0.001*	< 0.001*
LSD 5%	0.332	0.287	0.350	0.276	0.327

*Note:* Values within the same column sharing different letters are significantly different. Data are presented as mean ± SD, based on three replicates per group.

Abbreviations: CB, Coconut Butter; DB, Date Balls; PB, Peanut Butter; SB, Sesame Butter.

The superior consumer response to peanut butter formulations aligns with Shibli et al. (2019), who reported high acceptability of locally produced peanut butter based on flavor and texture. Likewise, El‐Enzi et al. (2018) observed that sesame flour fortification improved nutritional and sensory properties, while Benmeziane and Belleili (2023) found that coconut‐based milk products achieved favorable ratings due to their fatty acid composition and creamy mouthfeel. Our findings are also consistent with Asghar et al. (2021), who noted high acceptance for sweets prepared with dates and coconut paste, and with Yin et al. (2020), who highlighted the sensory appeal of sesame oils. More recently, studies by Hadi et al. (2023), Gichki et al. (2024), and Alsuhebani et al. (2025) confirmed the consumer preference for date‐based snacks such as bars and energy balls, especially as functional foods for athletes and health‐conscious populations.

Interestingly, coconut butter did not achieve the top acceptance level in this study, differing from the results of Raczyk et al. (2021), who reported higher consumer ratings for coconut‐enriched products. This discrepancy may be due to variations in regional taste preferences and familiarity with sesame products. Overall, the results confirm that enriching oat–date balls with peanut and coconut butters substantially improves sensory appeal, while sesame butter also provides acceptable sensory quality with the added benefit of nutritional enrichment.

### 3.6 Fatty Acid Profile of Formulated Oat‐Date Ball Oil Extractions

**FIGURE 2:**
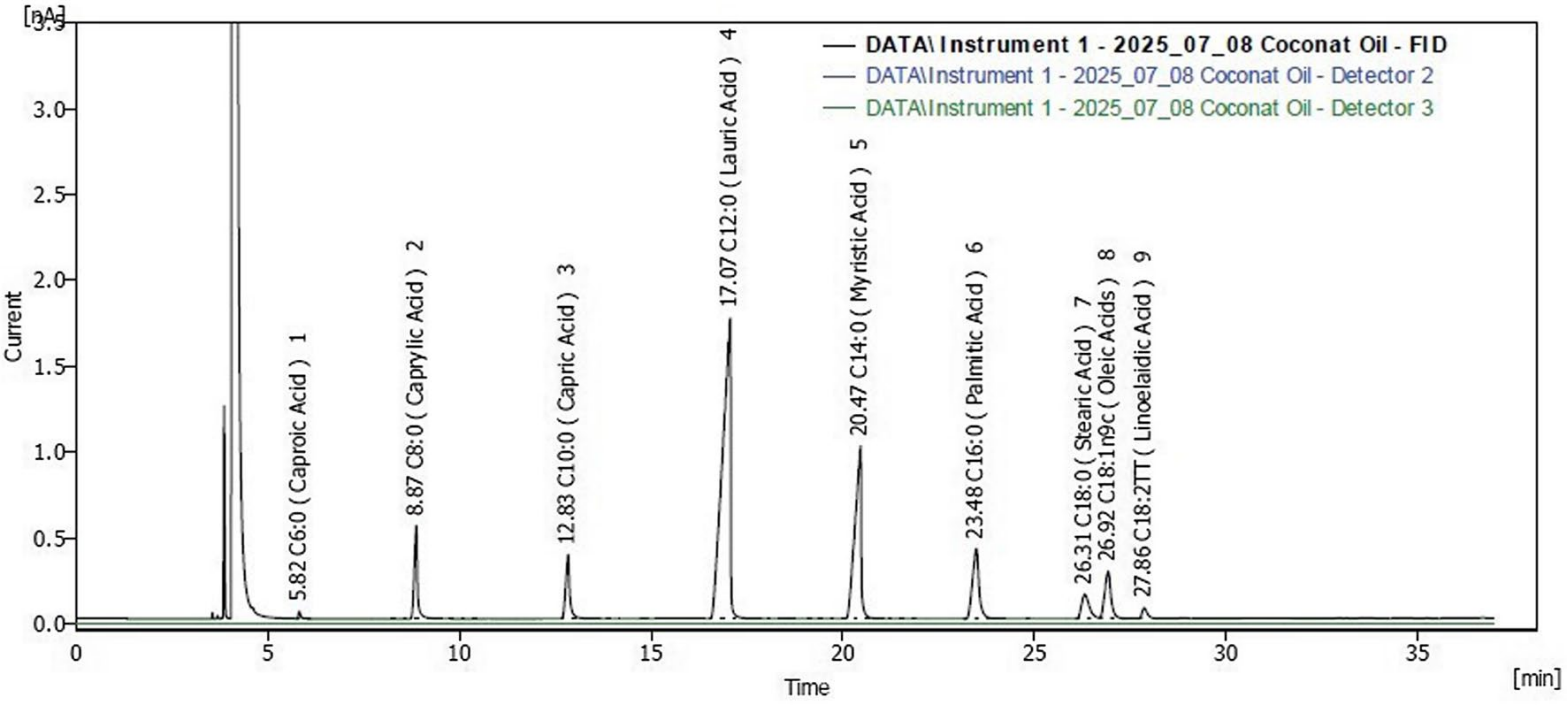
Fatty acid profile of formulated oat‐date ball coconut oil.

**FIGURE 3:**
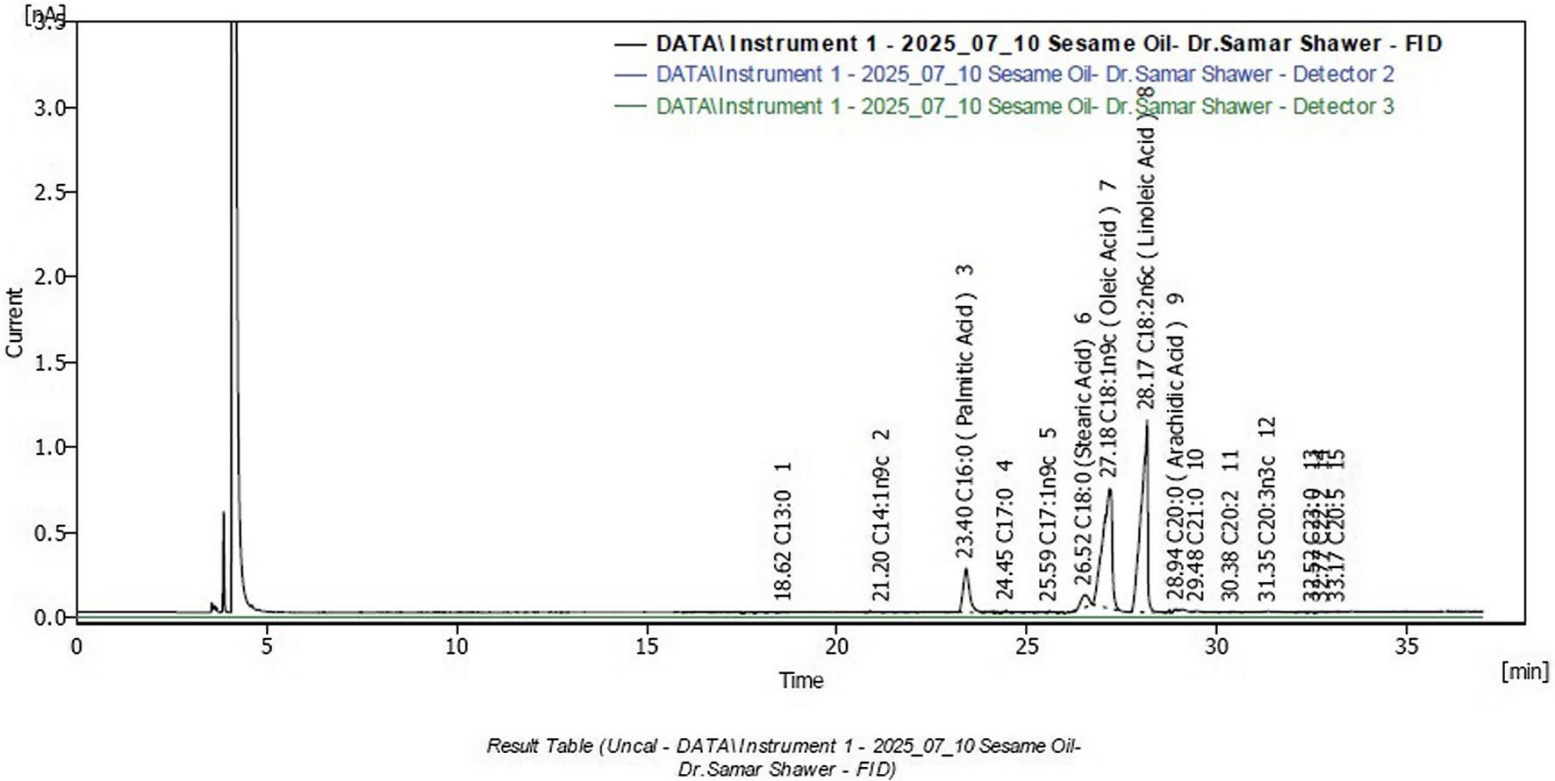
Fatty acid profile of formulated oat‐date ball sesame oil.

**FIGURE 4:**
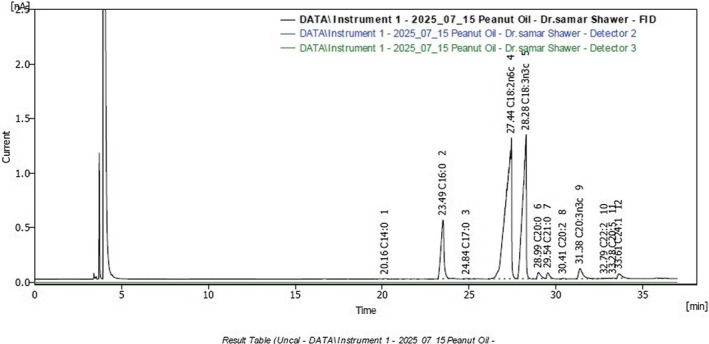
Fatty acid profile of formulated oat‐date ball peanut oil.

(Table 7) and Figure (2, 3, 4) present the fatty acid composition of oat‐date ball formulations. Date ball‐CB was dominated by saturated fatty acids (SFA, 93.6%), primarily lauric (C12:0, 48.1%), myristic (C14:0, 21.4%), and palmitic acids (C16:0, 9.0%), with notable medium‐chain fatty acids (MCFAs) such as caprylic (6.1%) and capric (5.2%), which are rapidly metabolized and contribute to oxidative stability and extended shelf life. This profile aligns with Dayrit (2015) and Perera et al. (2020), who identified lauric acid as the predominant component in coconut oil, and with Nitbani et al. (2022), who highlighted the antimicrobial and metabolic benefits of lauric acid and its monoglyceride, monolaurin (Figure 3).

**TABLE 7:** Fatty acid profile of formulated oat‐date ball coconut oil extractions, sesame oil extractions, and peanut oil extractions.

No.	Compound Name	Retention Time [min]	Area [%]
Coconut Oil			
1	C6:0 (Caproic Acid)	5.822	0.3
2	C8:0 (Caprylic Acid)	8.872	6.1
3	C10:0 (Capric Acid)	12.832	5.2
4	C12:0 (Lauric Acid)	17.067	48.1
5	C14:0 (Myristic Acid)	20.465	21.4
6	C16:0 (Palmitic Acid)	23.485	9.0
7	C18:0 (Stearic Acid)	26.308	3.5
8	C18:1n9c (Oleic Acid)	26.923	5.7
9	C18:2TT (Linolelaidic Acid)	27.860	0.8
Σ SFA			93.6
Σ MUFA			5.7
Σ PUFA			0.8
Total			100.0
Sesame Oil			
1	C13:0 (*Ginkgolic acid*)	18.622	1.0
2	C14:1n9c myristoleic acid	21.197	0.5
3	C16:0 (Palmitic Acid)	23.403	10.0
4	C17:0 Margaric acid	24.452	0.9
5	C17:1n9c margaroleic acid	25.592	0.9
6	C18:0 (Stearic Acid)	26.520	3.3
7	C18:1n9c (Oleic Acid)	27.175	38.5
8	C18:2n6c (Linoleic Acid)	28.167	42.5
9	C20:0 (Arachidic Acid)	28.937	0.3
10	C21:0 Heneicosanoic acid	29.480	0.2
11	C20:2 Eicosadienoic acid	30.383	0.3
12	C20:3n3cEicosatrienoic acid	31.353	0.6
13	C23:0Tricosanoic acid	32.522	0.5
14	C22:2Docosadienoic acid	32.767	0.3
15	C20:5Eicosapentaenoic acid	33.168	0.2
Σ SFA			16.2
Σ MUFA			39.9
Σ PUFA			43.9
Total			100
Peanut Oil			
1	C14:0 (Myristic acid)	20.162	0.1
2	C16:0 (Palmitic Acid)	23.493	11.0
3	C17:0 (Heptadecanoic acid)	24.845	0.1
4	C18:2n6c (Linoleic Acid)	27.437	50.9
5	C18:3n3c (Alpha‐Linolenic Acid)	28.280	31.9
6	C20:0 (Arachidic Acid)	28.990	1.3
7	C21:0 (Heneicosanoic acid)	29.538	1.0
8	C20:2 (Eicosadienoic acid)	30.405	0.1
9	C20:3n3c (Eicosatrienoic Acid)	31.378	2.6
10	C22:2 (Docosadienoic acid)	32.793	0.1
11	C20:5 (Eicosapentaenoic)	33.283	0.1
12	C24:1 (Nervonic acid)	33.613	1.0
Σ SFA			13.5
Σ MUFA			1.0
Σ PUFA			85.7
Total			100

Date ball–SB exhibited a highly unsaturated lipid profile, with PUFA (43.9%) and MUFA (39.9%) dominating and SFA at 16.2%. Linoleic acid (C18:2n6c, 42.5%) and oleic acid (C18:1n9c, 38.5%) were the major fatty acids, both associated with cardiovascular health. These results are broadly consistent with Devarajan et al. (2016), who reported comparable proportions of linoleic (41.28%) and oleic acids (42.91%) in sesame oil; although in the present study, linoleic acid slightly exceeded oleic acid. This difference may be attributed to varietal and extraction conditions. Supporting evidence from Wei et al. (2022) also confirms that sesame typically contains high levels of oleic and linoleic acids, reinforcing its nutritional value.

Date ball–PB displayed an even more unsaturated profile, dominated by PUFA (85.7%), with linoleic acid (50.9%) and α‐linolenic acid (31.9%) as major contributors, while SFA (13.5%) and MUFA (1.0%) were present in low amounts. This highly unsaturated composition is in agreement with Suchoszek‐Łukaniuk et al. (2011), who emphasized the cardiovascular benefits of high PUFA levels in peanut oil. Furthermore, Liu et al. (2025) reported that the abundant linoleic acid in peanut oil enhances its technological performance and sensory appeal in confectionery and snack products, which is consistent with our findings.

Overall, the fatty acid analyses confirm that each fat source contributes distinct nutritional and functional attributes: coconut butter offers oxidative stability through medium‐chain fatty acids (MCFAs), sesame butter provides a balanced unsaturation pattern supportive of heart health, and peanut butter delivers exceptionally high PUFA content, underscoring their collective potential as health‐promoting fat alternatives in functional snack formulations.

## 4 Conclusion

The incorporation of healthy plant‐based fat substitutes—coconut butter, sesame butter, and peanut butter—into oat–date balls significantly enhanced their nutritional composition, antioxidant activity, and sensory quality. Among the formulations, peanut butter contributed the highest protein enrichment and overall consumer acceptability, coconut butter provided the softest texture and highest energy value, while sesame butter offered the greatest mineral fortification, particularly in iron, calcium, and magnesium. All fat‐substituted samples demonstrated elevated phenolic and flavonoid contents, which translated into improved antioxidant capacity. Collectively, these results highlight the potential of plant‐based fats as functional ingredients in developing nutritionally balanced and consumer‐appealing snack products, especially for health‐conscious populations and those seeking natural alternatives to conventional fat sources.

## Author Contributions

Nashwa M. Younes was involved in conceptualization, validation, formal analysis, visualization, supervision, project administration, and funding acquisition. Mahmoud Younis was responsible for software support, resources, writing, review and editing, supervision, and funding acquisition. Mahmoud Khalil handled software, validation, formal analysis, investigation, resources, data curation, original draft preparation, visualization, supervision, and project administration. Diaeldin Omer Abdelkarim contributed to investigation, data curation, and writing, final revision. Samar Shawir contributed to validation, formal analysis, investigation, provision of resources, and writing, review and editing. All authors have read and approved the final version of the manuscript.

## Ethics Statement

All study experiments were ethically approved by the Scientific Research Ethics Committee, Faculty of Specific Education, Alexandria University (Approval no 07‐9‐2025; Serial Number: AU 0307487).

## Consent

All authors agree to publication.

## Conflicts of Interest

The authors declare no conflicts of interest.

## Data Availability

The data that support the findings of this study are available on request from the corresponding author. The data are not publicly available due to privacy or ethical restrictions.
